# Adverse events associated with molnupiravir: a real-world disproportionality analysis in food and drug administration adverse event reporting system

**DOI:** 10.3389/fphar.2023.1253799

**Published:** 2023-10-31

**Authors:** Yankun Liang, Lin Ma, Yuting Wang, Jingping Zheng, Ling Su, Jun Lyu

**Affiliations:** ^1^ Department of Clinical Research, The First Affiliated Hospital of Jinan University, Guangzhou, Guangdong, China; ^2^ School of Pharmaceutical Sciences, Jinan University, Guangzhou, Guangdong, China; ^3^ Guangdong Provincial Hospital of Traditional Chinese Medicine, Guangzhou, Guangdong, China; ^4^ Guangdong Provincial Key Laboratory of Traditional Chinese Medicine Information, Guangzhou, Guangdong, China

**Keywords:** molnupiravir, pharmacovigilance, coronavirus disease 2019, food and drug administration adverse event reporting system, adverse events, safety

## Abstract

Molnupiravir, an urgently approved drug during the Coronavirus Disease 2019 (COVID-19) pandemic, serves as the basis for our study, which relies on the Food and Drug Administration Adverse Event Reporting System (FAERS). The objective is to extract adverse event (AE) signals associated with molnupiravir from the FAERS database, thereby providing a reference for post-marketing monitoring of adverse events. Specifically, we extracted individual case safety reports (ICSRs) from the database, focusing on cases with COVID-19 indications and molnupiravir identified as the primary suspect drug. Descriptive analysis of the extracted data was performed, followed by four disproportionality analyses using the reporting odds ratio (ROR) method. These analyses were conducted across four levels, encompassing overall data, reports by health professionals, as well as age and gender differentiations, ensuring the robustness of the analysis results. In total, 116,576 ICSRs with COVID-19 indications and 2,285 ICSRs with molnupiravir as the primary suspect were extracted. Notably, after excluding cases with unknown age or gender, a higher proportion of molnupiravir-related ICSRs were observed among individuals aged 65 years and older (70.07%) and women (54.06%). The most frequently reported adverse events and AE signals were associated with gastrointestinal disorders, as well as skin and subcutaneous tissue disorders. Moreover, individuals aged 65 years and older exhibited a higher risk of cardiac disorders, hepatobiliary disorders, renal and urinary disorders, and vascular disorders. In conclusion, this study found molnupiravir demonstrated a lower risk of serious adverse events compared to other RNA antiviral drugs like remdesivir in patients under 65 years old. However, close monitoring of its safety is still necessary for elderly patients aged 65 years and above. Further studies are warranted to continuously assess the safety profile of molnupiravir as its usage increases, especially in high risk populations.

## 1 Introduction

The Coronavirus Disease 2019 (COVID-19) pandemic caused by Severe Acute Respiratory Syndrome Coronavirus 2 (SARS-CoV-2) is a highly infectious disease, with a rapid person-to-person transmission rate. According to the WHO Coronavirus (COVID-19) Dashboard, as of 10 February 2023, there have been 760 million confirmed cases of COVID-19 worldwide, resulting in 6.86 million deaths (World Health Organization, 2023). COVID-19 can range from asymptomatic to severe respiratory failure and multi-organ involvement (Huang et al., 2020; Wang et al., 2020). The major symptoms of COVID-19 include fever, cough, and dyspnea (De Vito et al., 2021; Cascella et al., 2023). The minor symptoms are less specific and require comprehensive evaluation. They include anosmia/dysgeusia, headache, diarrhea, vomiting, nausea, sore throat, fatigue, malaise, myalgia, etc (Cheung et al., 2020; Grant et al., 2020; Mao et al., 2020; Vaira et al., 2020). Skin lesions such as vasculitis-like skin eruption can also be seen in COVID-19 patients (Geremia et al., 2020). Although vaccination has significantly reduced the risk of morbidity and mortality (Polack et al., 2020), breakthrough infections remain a concern. Before molnupiravir, remdesivir was the first antiviral against SARS-CoV-2, sharing the same target as molnupiravir. Initially, remdesivir was administered to patients with severe pneumonia and illness (De Vito et al., 2022), but following the PINE-TREE trial, it was also recommended for mild cases to prevent progression (Gottlieb et al., 2022). However, remdesivir requires intravenous infusion, making an oral alternative highly desirable.

Targeting the RNA-dependent RNA polymerase (RdRp), which is a crucial enzyme for SARS-CoV-2 replication, has been proven to be an effective strategy to combat COVID-19, regardless of the variant type. This is because RdRps are widely conserved among all SARS-CoV-2 strains (Rabie, 2022). Molnupiravir is a biologically active prodrug of b-D-N4-hydroxycytidine (NHC, EIDD-1931) that targets RdRp. Upon oral administration, molnupiravir is quickly converted into active NHC in plasma and distributed to various organs. Host kinases then convert it into NHC 5′-triphosphate. This NHC 5′-triphosphate can serve as a competitive alternative substrate for viral RdRp, which integrates into viral RNA and causes the accumulation of mutations in the viral genome, ultimately leading to lethal mutations (Tian et al., 2022). In the MOVE-OUT trial, a phase 3, double-blind, randomized, placebo-controlled trial, molnupiravir demonstrated a 31% relative risk reduction in all-cause mortality compared to placebo. Additionally, the proportion of participants experiencing at least one adverse event was similar in both the molnupiravir and placebo groups, indicating its safety (Jayk Bernal et al., 2022). Being a potential therapeutic option, molnupiravir received emergency use authorization from the Food and Drug Administration (FDA) on 23 December 2021, for the management of mild-to-moderate COVID-19 in adults who are at a heightened risk of developing severe illness (CDER Division of Drug Information, 2023). In addition to the United States, molnupiravir was granted approval in the United Kingdom in November 2021. Furthermore, it received special emergency approval in Japan on 24 December 2021 (Medicines and Healthcare products Regulatory Agency, 2021; Merck & Co, I, 2021). However, the PANORAMIC trial showed no benefits of molnupiravir over placebo in reducing mortality or hospitalization duration for hospitalized COVID-19 patients (Butler et al., 2023). Additionally, the European Medicines Agency’s controversial withdrawal of molnupiravir’s regulatory application (EMA, 2023) has raised doubts about its efficacy. While the efficacy of molnupiravir remains questionable given these latest trial results and regulatory decisions, some advantages of molnupiravir’s safety profile should be considered. Specifically, unlike other antiviral options like remdesivir and nirmatrelvir, molnupiravir has demonstrated a lower potential for clinically significant drug-drug interactions (DDIs) so far (Wanounou et al., 2022; Akhvlediani et al., 2023). The low DDI risk makes molnupiravir an easier oral therapy to administer alongside other medications patients may be taking. Therefore, if the efficacy concerns can be adequately addressed with additional studies, molnupiravir’s favorable DDI profile could position it as an alternative oral antiviral option, especially for patients on polypharmacy regimens.

Molnupiravir has not only demonstrated a comparable incidence of adverse reactions in both the molnupiravir and placebo groups during the MOVE-OUT trial but also consistently across all current clinical trials at doses of 200 mg, 400 mg, and 800 mg (Caraco et al., 2022; Fischer et al., 2022; Sinha et al., 2022; Tippabhotla et al., 2022; Zou et al., 2022; Butler et al., 2023; Khoo et al., 2023). Moreover, systematic reviews and meta-analyses suggest that molnupiravir may represent a safe and effective treatment option for patients with COVID-19 (Joseph Mari and Erwin, 2022; Mali et al., 2022; Pitre et al., 2022). Although current clinical trials and systematic reviews indicate that molnupiravir appears to have a favorable safety profile, it is important to note that the safety outcomes in these trials are typically assessed over a limited period of 28–29 days. Therefore, there is still a lack of comprehensive real-world research and post-marketing monitoring for this drug. To address this critical gap, our study aims to gather individual case safety reports associated with molnupiravir from the FDA Adverse Event Reporting System (FAERS) database. By analyzing these reports, we intend to identify potential adverse drug reactions associated with molnupiravir and compare them with other SARS-CoV-2 RNA drugs such as remdesivir, ribavirin, favipiravir, and azvudine. This study aims to contribute to the post-marketing monitoring of molnupiravir and provide valuable insights for its clinical use.

## 2 Materials and methods

FAERS is a computerized database specifically designed for the spontaneous reporting of adverse events and medication errors involving human drugs and therapeutic biological products. The data structure of FAERS adheres to the International Council for Harmonisation (ICH) guidelines for international safety reporting. Adverse events and therapeutic indications are coded at the “preferred term” (PT) level using the Medical Dictionary for Regulatory Activities (MedDRA).

Access to FAERS data is provided through quarterly data files, which can be obtained from the following link: https://fis.fda.gov/extensions/FPD-QDE-FAERS/FPD-QDE-FAERS.html. These files are available in two distinct formats: ASCII files and XML files. For our study, we downloaded FAERS data in ASCII format, covering the period from January 2020 to December 2022. We managed and analyzed the data using Microsoft SQL Server 2019 software. Since the study was an analysis of the third party anonymized publicly available database with pre-existing institutional review board (IRB) approval, IRB approval was exempted by the institutional ethics board of The first Affiliated Hospital Of Jinan University.

To prevent the duplication of multiple report versions, we conducted a deduplication process on the DEMO table. Firstly, we removed identical records, keeping only one instance. Next, if the “CASEID” column was the same, we deleted the duplicate “PRIMARYID” column with the lower value. Additionally, if multiple rows had the same “PRIMARYID” values, we eliminated the earliest “FDA_DT” column to ensure data consistency (Silva et al., 2021).

In order to investigate the adverse event (AE) signals of molnupiravir in COVID-19 prevention and treatment, we utilized the following narrow Standardized MedDRA Query (SMQ) within the “INDI” table: COVID-19, COVID-19 pneumonia, COVID-19 immunization, COVID-19 prophylaxis, COVID-19 treatment, Suspected COVID-19, Asymptomatic COVID-19, SARS-CoV-2 test positive, SARS-CoV-2 carrier, SARS-CoV-2 test false negative, SARS-CoV-2 antibody test positive, SARS-CoV-2 sepsis, SARS-CoV-2 viremia, Occupational exposure to SARS-CoV-2, Exposure to SARS-CoV-2, Coronavirus infection, Coronavirus test positive, Multisystem inflammatory syndrome in children. These queries were employed to extract reports involving COVID-19 from the FAERS database (Wu et al., 2022).

We proceeded to identify cases in the “DRUG” table where the “drugname” and “prod_ai” columns aligned with the regular expression "%MOLNUPIRAVIR%" or "% LAGEVIRIO%", and the “role_cod” column matched the regular expression “PS”. These cases represented instances where molnupiravir was administered.

Initially, we examined the attributes of the included Individual Case Safety Reports (ISCRs), which encompassed age, gender, reporter type, reporting country, serious outcomes and dosage. The serious outcomes evaluated encompassed death, life-threatening conditions, interventions, disabilities, congenital anomalies, hospitalizations, and other significant events.

We proceeded with a detailed analysis of the attributes of the ISCRs. Furthermore, a disproportionality analysis was conducted to detect any potential AE signals. To ensure the reliability of the findings, separate disproportionality analyses were performed based on patient age and sex. Additionally, a sensitivity analysis was conducted by restricting the analysis to reports from healthcare professionals as the reporters. In our study, adverse event signals such as “product use issue,” “no adverse event,” “wrong technique in product usage process,” “COVID-19”and similar cases were excluded to enhance the reliability of the findings.

The descriptive statistical analysis encompassed patient age, gender, reporter type, reporting country, dosage and the outcomes of adverse events. The variables are presented as frequencies and percentages.

For the disproportionality analysis, we employed the reporting odds ratio (ROR) method and conducted calculations using a 2-by-2 contingency table (Table 1). In this analysis, reports where molnupiravir was suspected as the causative drug were classified in the target drug group, while reports without molnupiravir as the suspected drug were included in the other drug group. A risk signal was deemed significant if the total number of drugs and adverse events was three or more, and the lower limit of the 95% confidence interval (CI) for the ROR exceeded 1 (Jung et al., 2021; Kim et al., 2021; Rocca et al., 2021).

$$ROR=\frac{a/c}{b/d}=\frac{ad}{bc}$$

$$ROR95\%CI=e^{\ln\left(ROR\right)\pm 1.96\sqrt{\left(\frac{1}{a}+\frac{1}{b}+\frac{1}{c}+\frac{1}{d}\right)}}$$

**TABLE 1:** 2-by-2 contingency table.

	Target adverse event	Other adverse event	Sums
Molnupiravir	a	b	a + b
Other drugs	c	d	c + d
Sums	a + c	b + d	N = a + b + c + d

## 3 Results

### 3.1 Data extraction

**FIGURE 1:**
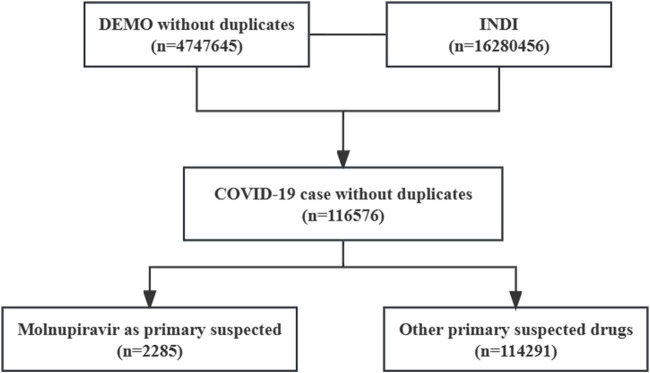
Individual case safety reports identification process.

A comprehensive analysis was conducted on a dataset of 4,747,645 individual case safety reports (ICSRs) from the FAERS database, spanning January 2020 to December 2022, after removing duplicates. Among them, 116,576 ICSRs indicated COVID-19. For the analysis, 2,285 ICSRs were included in the molnupiravir group, while 114,291 ICSRs were assigned to the other drugs group. More information on ICSR identification can be found in Figure 1.

### 3.2 Descriptive analysis

**FIGURE 2:**
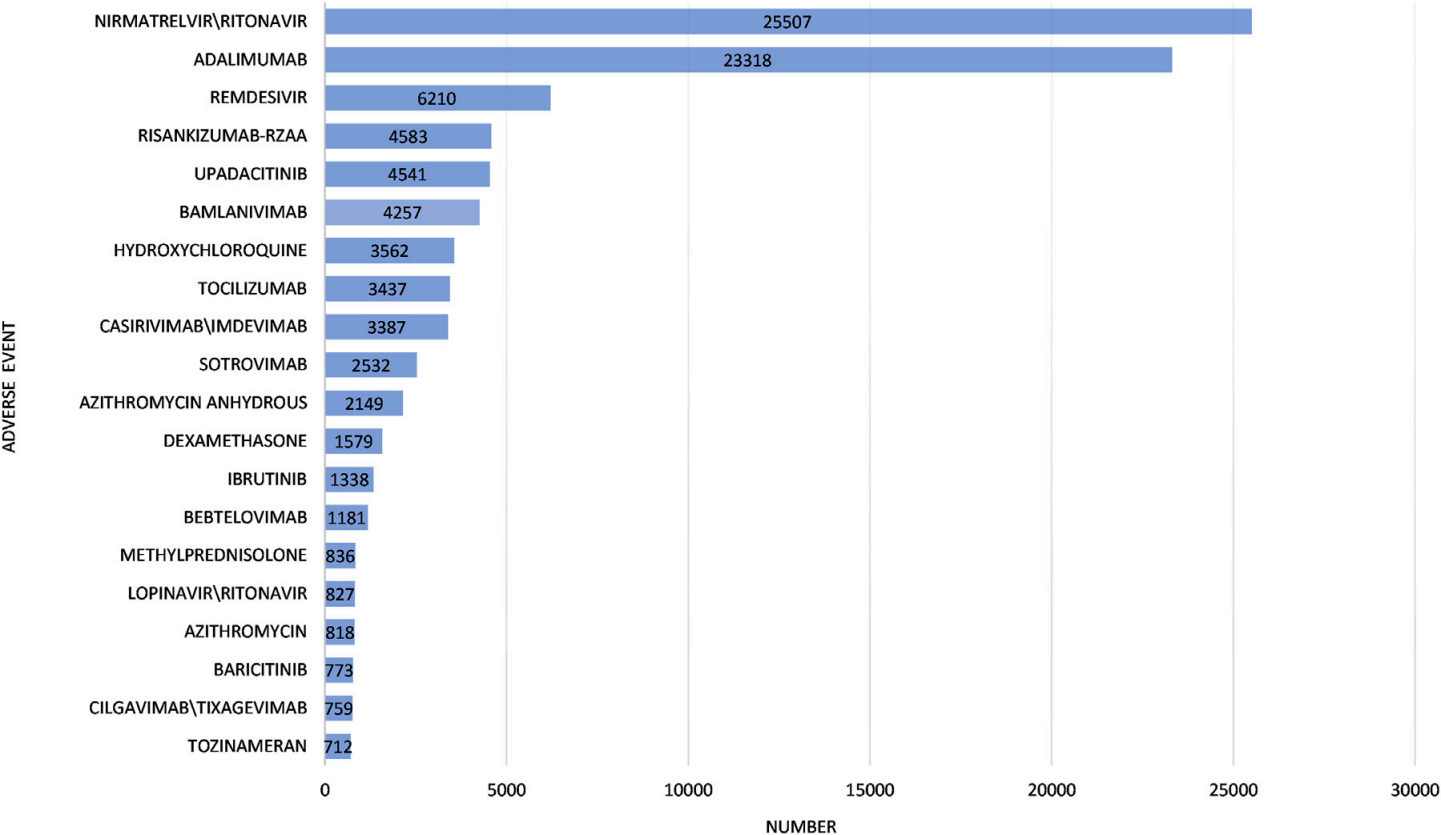
Top 20 drugs involved in adverse events in the other Coronavirus Disease 2019 drugs group.

The characteristics of the 116,576 ICSRs are presented in Table 2. Notably, the molnupiravir group exhibited a higher proportion (60.96%) of individuals aged 65 years and older, compared to the other drugs group (27.42%). The male-to-female ratio was 0.85 in the molnupiravir group and 0.75 in the other drugs group. Assessing outcomes, the other drugs group reported a higher incidence of life-threatening events (3.40% vs. 2.19%), interventions (0.94% vs. 0.70%), and hospitalizations (20.14% vs. 19.65%) compared to the molnupiravir group. Conversely, the molnupiravir group reported a higher rate of deaths (12.12% vs. 6.99%) compared to the other drugs group. The top 20 drugs involved in adverse events in the other drugs group are summarized in Figure 2.

**TABLE 2:** The characteristics of the 116576 COVID-19 ICSRs.

Characteristics	MOLNUPIRAVIR (N = 2,285)		Other COVID-19 drugs (N = 114,291)	
	N	%	N	%
Age (years)				
<18	24	1.05%	1,327	1.16%
18–44	150	6.56%	14,604	12.78%
45–64	421	18.42%	27,827	24.35%
≥65	1,393	60.96%	31,339	27.42%
Unknown	297	13.00%	39,194	34.29%
Sex				
Female	1,139	49.85%	59,484	52.05%
Male	968	42.36%	44,563	38.99%
Unknown	178	7.79%	10,244	8.96%
Type of reporter				
Health professional	1830	80.09%	30,541	26.72%
Non-Health professional	307	13.44%	62,503	54.69%
Unknown	148	6.48%	21,247	18.59%
Reporting country				
United States	360	15.75%	78541	68.72%
Japan	1,667	72.95%	2,717	2.38%
Other countries	258	11.29%	24,466	21.41%
Not Specified	0	0.00%	8,567	7.50%
Outcome				
Death	277	12.12%	7,989	6.99%
Life Threatening	50	2.19%	3890	3.40%
Required Intervention	16	0.70%	1,070	0.94%
Disabled	19	0.83%	961	0.84%
Hospitalizations	449	19.65%	23,021	20.14%
Congenital Anomaly	0	0.00%	118	0.10%
Other Outcomes	701	30.68%	40,316	35.27%
Non-Serious	773	33.83%	36,926	32.31%
Dosage				
Dosage according to the label	1,444	63.19%	\	\
Dosage below the label	201	8.80%		
Dosage above the label	32	1.40%		
Unknown	608	26.61%		

**FIGURE 3:**
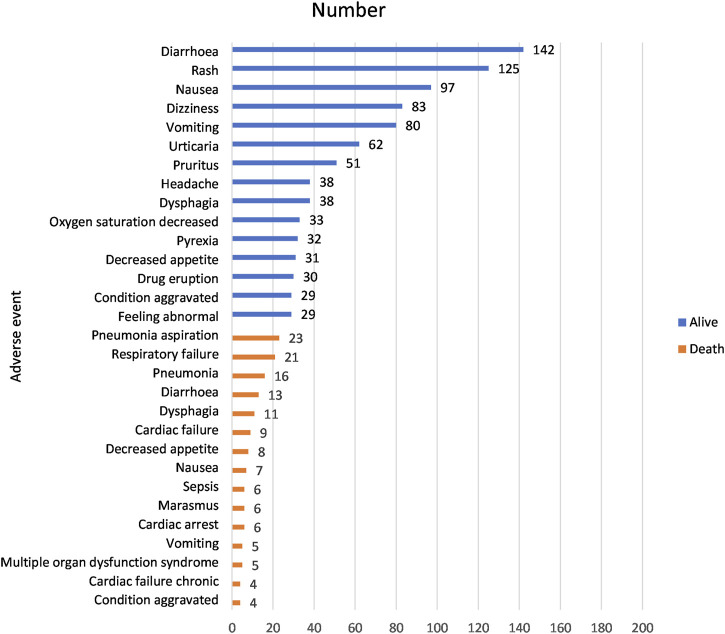
Most common adverse events associated with the use of molnupiravir.

The molnupiravir group included 2,285 individual safety case reports (ISCRs) encompassing a total of 4,888 adverse events, while the non-molnupiravir group comprised 114291 ISCRs involving 374575 adverse events. We identified the top 15 AEs based on occurrence and deaths. The most common AEs were Diarrhoea (142, 2.91%), Rash (125, 2.56%), Nausea (97, 1.98%), Dizziness (83, 1.70%) and Vomiting (80, 1.64%); AEs leading to death included, Pneumonia aspiration (23, 0.47%), Respiratory failure (21, 0.43%), Pneumonia (16, 0.33%), Diarrhoea (13, 0.27%) and Dysphagia (11, 0.23%) (Figure 3).

### 3.3 Disproportionality analysis

#### 3.3.1 Overall

The molnupiravir group comprised 2,285 ICSRs. Using the ROR method, 74 AE signals were identified and classified into 18 System Organ Classes (SOCs). The top 20 AE signals at the preferred term PT level are shown in Table 3. They cover 9 SOCs, with skin/subcutaneous tissue disorders and gastrointestinal disorders (GI) being the most common.

**TABLE 3:** The top 20 AE signals at the PT level.

SOC	AE	N	ROR (95%CI)
Skin and subcutaneous tissue disorders (227)	Drug eruption	30	37.30 (24.10–57.74)
	Toxic skin eruption	10	33.38 (15.88–70.18)
	Rash	125	4.51 (3.76–5.41)
	Urticaria	62	4.47 (3.45–5.78)
Gastrointestinal disorders (73)	Diverticulum intestinal haemorrhagic	3	57.51 (12.87–257.02)
	Faeces soft	9	12.12 (6.00–24.49)
	Melaena	12	10.47 (5.73–19.16)
	Dysphagia	49	6.86 (5.12–9.20)
Infections and infestations (44)	Pneumonia aspiration	44	30.92 (21.77–43.91)
Respiratory, thoracic and mediastinal disorders (19)	Sputum retention	5	63.92 (19.50–209.53)
	Aspiration	8	16.59 (7.72–35.65)
	Lower respiratory tract congestion	3	12.11 (3.58–40.93)
	Asphyxia	3	11.50 (3.42–38.72)
Nervous system disorders (14)	Altered state of consciousness	7	10.53 (4.78–23.22)
	Cerebral infarction	7	8.80 (4.03–19.26)
Psychiatric disorders (13)	Hallucination, visual	8	13.95 (6.57–29.66)
	Abnormal behaviour	5	10.37 (4.07–26.38)
Renal and urinary disorders (12)	Urinary retention	12	7.81 (4.31–14.15)
Metabolism and nutrition disorders (6)	Marasmus	6	153.45 (38.37–613.75)
Cardiac disorders (4)	Cardiac failure chronic	4	15.34 (5.24–44.89)

According to the drug labels, the most common adverse events associated with molnupiravir use are diarrhea, nausea, and dizziness, which align with the findings from our analysis (MSD LLC, 2023). These adverse events have been included in our monitoring list. In addition to the adverse events mentioned in the drug labels, we have detected 71 AE signals that are not specifically mentioned.

#### 3.3.2 Reported by health professionals

To ensure reliability, we extracted ICSRs reported by health professionals (physicians, pharmacists, and other healthcare professionals) from the initial pool of 2,285 ICSRs associated with molnupiravir. The health professional group consisted of 1,830 ICSRs related to molnupiravir. Using the ROR method, we identified 52 AE signals in the health professional group. The top 20 AE signals at the PT level are presented in Table 4.

**TABLE 4:** The top 20 AE signals at the PT level of the health professional group.

SOC	AE	N	ROR (95%CI)
Gastrointestinal disorders (176)	Faeces soft	7	19.06 (7.10–51.22)
	Dysphagia	42	8.95 (6.28–12.76)
	Diverticulum intestinal haemorrhagic	3	36.73 (6.13–219.88)
	Haematochezia	11	10.79 (5.31–21.95)
	Diarrhoea	113	3.63 (2.97–4.43)
Skin and subcutaneous tissue disorders (132)	Drug eruption	29	28.60 (16.73–48.88)
	Toxic skin eruption	9	10.51 (4.81–22.96)
	Dermatitis allergic	4	16.33 (4.61–57.88)
	Rash	81	3.98 (3.14–5.04)
	Eczema	9	5.52 (2.67–11.38)
Infections and infestations (44)	Pneumonia aspiration	44	16.50 (11.25–24.20)
Metabolism and nutrition disorders (12)	Marasmus	6	49.01 (12.25–196.05)
	Feeding disorder	6	8.17 (3.24–20.59)
Psychiatric disorders (11)	Hallucination, visual	7	9.03 (3.79–21.49)
	Abnormal behaviour	4	8.91 (2.83–27.98)
Renal and urinary disorders (11)	Urinary retention	11	10.79 (5.31–21.95)
Respiratory, thoracic and mediastinal disorders (8)	Sputum retention	5	61.25 (11.88–315.80)
	Asphyxia	3	12.24 (3.06–48.97)
Nervous system disorders (7)	Altered state of consciousness	7	6.86 (2.97–15.87)
Ear and labyrinth9 disorders (4)	Deafness unilateral	4	32.66 (7.31–145.96)

#### 3.3.3 Age

**FIGURE 4:**
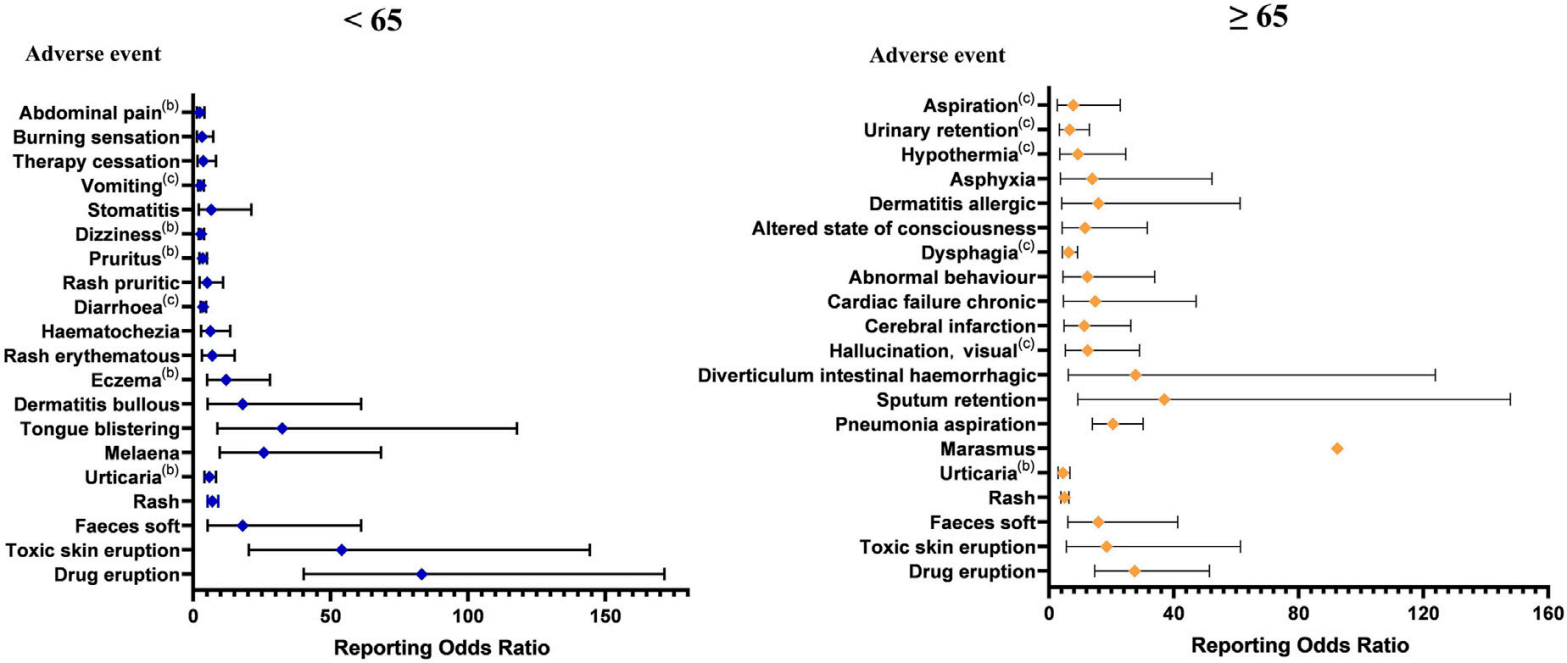
The top 20 adverse event signals at the preferred term level base on age. (a The adverse event is up to level 2 in Common Terminology Criteria for Adverse Events. b The adverse event is up to level 3 in Common Terminology Criteria for Adverse Events. c The adverse event is up to level 5 in Common Terminology Criteria for Adverse Events.).

Of the 2,285 ICSRs associated with molnupiravir, 2,042 reported known age and were divided into <65 years and ≥65 years groups. The remaining 243 ICSRs had unknown age. The <65 years group had 595 ICSRs, while the ≥65 years group had 1,393 ICSRs. Using the ROR method, we identified 30 AE signals in the <65 years group and 56 AE signals in the ≥65 years group. It is worth noting that only five signals were common among the top 20 signals in the two age groups. According to Common Terminology Criteria for Adverse Events (CTCAE), in <65 years group, 5 PTs were Grade 3 and 2 PTs were Grade 5 among top 20 adverse events at PT level. In ≥65 years group, 1 PT was Grade 3 and 5 PTs were Grade 5, excluding those not covered by CTCAE (U.S. DEPARTMENT OF HEALTH AND HUMAN SERVICES, 2017). Overall, adverse events appeared to be more severe in the <65 years group (Figure 4).

#### 3.3.4 Sex

**FIGURE 5:**
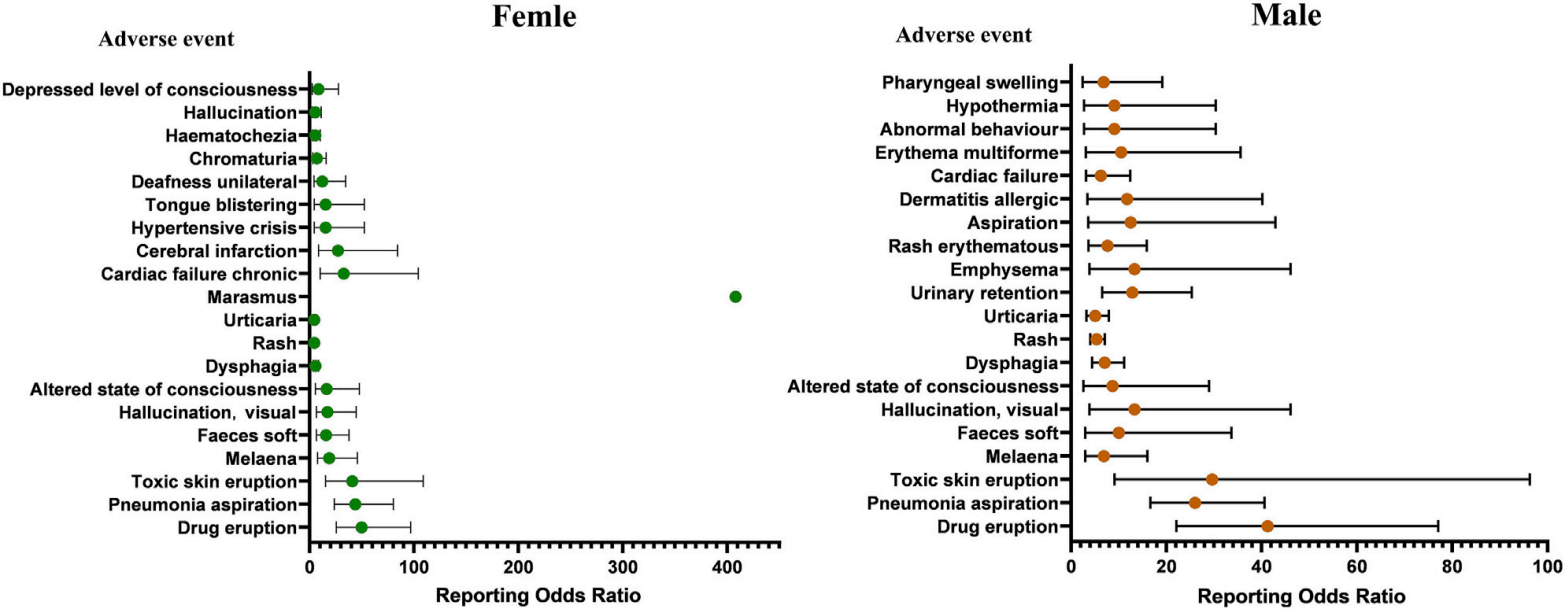
The top 20 adverse event signals at the preferred term level base on sex.

In addition to age sensitivity analysis, we also conducted a sex sensitivity analysis. Of the 2,285 ICSRs associated with molnupiravir, 2,107 reported known gender and were divided into female (1,139 ICSRs) and male (968 ICSRs) groups. The remaining 178 ICSRs had unknown gender. Using ROR, we found 54 AE signals in the female group and 43 AE signals in the male group. RORs with 95% CIs were calculated for each group, and the top 20 adverse drug event signals at the PT level are listed in Figure 5.

It is noteworthy that marasmus (ROR: 408.07, 95% CI: 47.66–3494.22) emerged as the strongest adverse drug event signal in the female group, but it did not appear among the top 20 adverse drug event signals in the male group. The mean time to marasmus onset was 1.75 ± 1.71 days in the molnupiravir group and 16.50 ± 10.60 days in the other COVID-19 drugs group, *p* = 0.001.

## 4 Discussion

Molnupiravir, an RNA drug targeting SARS-CoV-2, is being utilized on an emergency basis in numerous countries for the treatment of COVID-19. Various studies have suggested that molnupiravir could be a secure therapeutic option to lower hospitalizations and/or mortality rates among nonhospitalized individuals with COVID-19 (Gao et al., 2023). However, the presence of certain unknown concerns, including teratogenicity, carcinogenicity, mutation, and potential bone and cartilage toxicity, cannot be overlooked (Focosi, 2022). Currently, there is a scarcity of studies investigating the long-term toxicity of molnupiravir. Most of these studies are prospective in nature and have small sample sizes, while there is a lack of retrospective studies with large sample cohorts. In our research, we conducted the first largescale retrospective study utilizing the FAERS database to examine adverse events associated with molnupiravir. Our findings indicate that molnupiravir is associated with an elevated likelihood of gastrointestinal disorders and skin and subcutaneous tissue disorders as adverse events. Moreover, we observed a significant increase in the risk of AEs related to cardiac disorders, hepatobiliary disorders, renal and urinary disorders, and vascular disorders among individuals aged 65 years and older. However, there was no statistically significant difference observed between males and females.

The characteristics of individual safety reports linked to molnupiravir highlight that individuals aged 65 years and older account for over 60% (60.96%) of the reported cases. This observation may be attributed to the approved usage scope of molnupiravir, such as in the United States where LAGEVRIO™ (molnupiravir) is an investigational medicine for treating mild to moderate COVID-19 in adults who have a positive result from a direct-to-consumer SARS-CoV-2 virus test and are at risk of progressing to severe COVID-19. These individuals are typically unable to access or receive clinically suitable FDA-approved or licensed COVID-19 treatment options. Adolescents, on the other hand, tend to possess robust immune systems and generally experience milder illness following severe acute respiratory syndrome coronavirus 2 infection. Additionally, elderly individuals and those at higher risk often receive chronic treatments that may interact with Nirmatrelvir/ritonavir, making molnupiravir a more suitable option for them. The substantial number of elderly individuals using molnupiravir has contributed to the multitude of individual case safety reports, which is one of the factors we suspect. Diarrhea, nausea, and dizziness are the most frequently reported adverse drug events associated with molnupiravir treatment in most clinical trials, aligning closely with the outcomes of our analysis on the common adverse events linked to molnupiravir usage (Sinha et al., 2022). This study validates the effectiveness of utilizing a data mining method for detecting AE signals, making it a valuable reference for clinical applications. However, it should be noted that diarrhea and rash can also manifest as common symptoms of COVID-19 itself, making it difficult to ascertain whether they are truly drug-related adverse events or merely symptoms of the viral infection.

The results of the disproportionality analysis for the molnupiravir group and health professional group showed that adverse events mainly linked to molnupiravir were related to GI disorders and skin/subcutaneous tissue disorders. We found 71 adverse event signals not mentioned in the drug label, with the majority involving skin/subcutaneous tissue disorders. In a phase I, randomized, placebo-controlled study with healthy Japanese participants, toxic skin eruption was the most frequently reported adverse event associated with molnupiravir use (Nakamura et al., 2022). In another trial, a similar observation was made where discontinuation of the treatment occurred due to a rash in one participant (Painter et al., 2020). Skin and subcutaneous tissue disorder adverse events were infrequent in some COVID-19 patient studies. However, the ethnic sensitivity of these findings remains unclear due to the limited scale of our study. Continuous surveillance is crucial to further investigate and monitor these aspects effectively.

**FIGURE 6:**
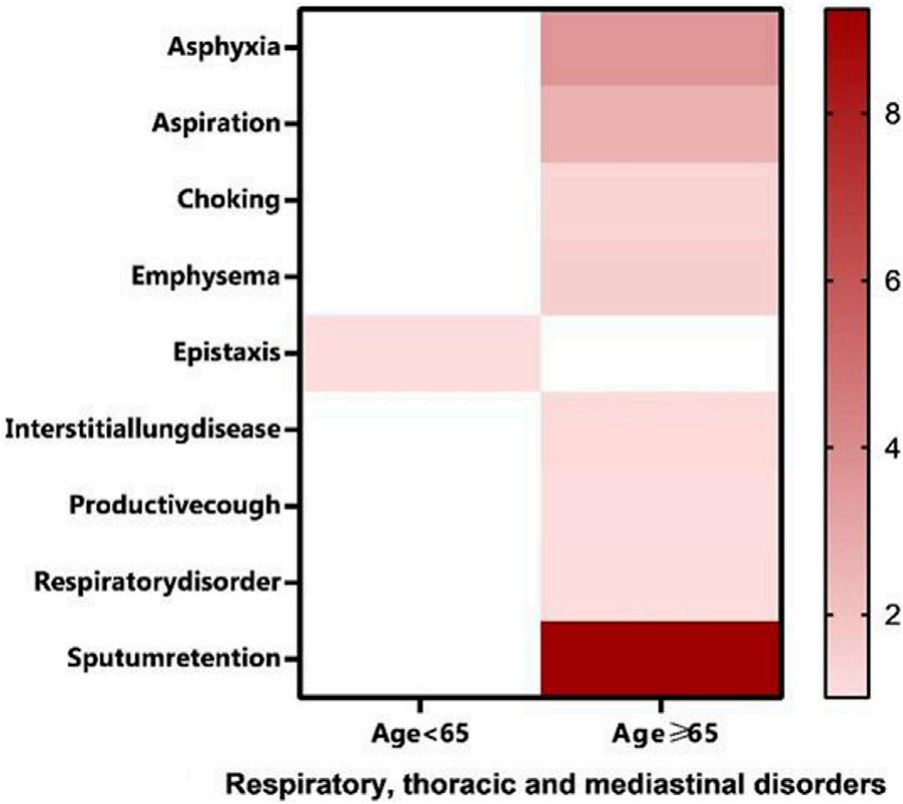
Heat map of Respiratory, thoracic and mediastinal disorders.

Close monitoring of individuals aged ≥65 years is recommended to mitigate serious adverse reactions with molnupiravir. Among the AE signals, 30 were observed in the <65 years group, while 56 were reported in the ≥65 years group, involving 9 SOCs and 14 SOCs, respectively. Notably, the four SOCs related to cardiac disorders, hepatobiliary disorders, renal and urinary disorders, and vascular disorders were exclusively present in the ≥65 years group. These SOCs are associated with more severe PTs not mentioned in the drug labels, such as chronic cardiac failure, abnormal hepatic function, urinary retention, and hypertensive crisis. Additionally, certain clinical trials have reported serious adverse events like cardiac chest pain, haematuria, hypertension, and increased transaminase levels, which are not included in the molnupiravir drug labels (Painter et al., 2020; Fischer et al., 2022). Therefore, increased surveillance of cardiotoxicity, nephrotoxicity, hepatotoxicity, and vascular disease is warranted in elderly patients. Additionally, notable differences exist between the two age groups in the respiratory, thoracic, and mediastinal disorders system. To enhance clarity, we created a heat map (Figure 6). The ≥65 years group showed more signals in respiratory, thoracic, and mediastinal disorders, with the strongest signal being sputum retention (ROR: 36.97, 95% CI: 9.24–147.88). Hence, middle-aged and elderly individuals may require heightened monitoring of adverse events, particularly for cardiac disorders, hepatobiliary disorders, renal and urinary disorders, vascular disorders, and the respiratory, thoracic, and mediastinal disorders system. We also found females ≥65 years old had a higher risk of developing marasmus. Possible mechanisms include appetite and metabolic changes, and direct drug toxicity. Further studies on pathophysiology are warranted. In the interim, clinicians should closely monitor nutritional status and wasting in molnupiravir patients, especially elderly females in the first week. Prompt nutrition and physical therapy support may mitigate progression. The risk of rapid severe marasmus onset should be considered when evaluating molnupiravir’s risk-benefit profile.

Molnupiravir has demonstrated excellent safety as an SARS-CoV-2 RNA drug. In comparison, other SARS-CoV-2 RNA drugs have exhibited distinct adverse events in pharmacovigilance studies based on real FAERS database. Remdesivir has shown a significant association with acute kidney injury, with the top three adverse events being elevated liver function test, acute kidney injury, and death. The ribavirin-interferon combination has been linked to an increased risk of anemia, vomiting, neutropenia, diarrhea, and insomnia. Favipiravir has shown side effects such as QTC prolongation, hyperuricemia, abnormalities in liver enzymes, elevation of uric acid, total bilirubin, and liver enzymes, along with gastrointestinal disorders in clinical trials. Conversely, no adverse events were reported in the azvudine group in a clinical trial (Ren et al., 2020; Shan et al., 2020; Wu et al., 2022; Alsuhaibani et al., 2023; Batool et al., 2023). In addition, clinical trials have indicated that Molnupiravir is associated with fewer side effects compared to nirmatrelvir/ritonavir (Mazzitelli et al., 2023). Although GI disorders and skin/subcutaneous tissue disorders are the most common AEs related to molnupiravir, it is essential to enhance safety monitoring in older and medically vulnerable individuals.

Our study has limitations. Firstly, as a spontaneous reporting system, the FAERS database lacks information on patients’ baseline characteristics (e.g., age, gender, BMI), onset timing AE severity and so on. These factors limit the predictive ability and detail of our analysis. Secondly, the substantially larger sample size of the non-molnupiravir group compared to the molnupiravir group may lead to baseline imbalance that cannot be readily addressed due to the inherent data source discrepancy. We mention that this could be a limitation in result interpretation. Furthermore, FAERS does not contain information on patient ethnicity, making it impossible to analyze potential differences in adverse events between populations such as Japanese versus Caucasians. The lack of ethnicity data is an important limitation, as previous studies have shown pharmacokinetic differences between ethnic groups (Shimada et al., 1994). In addition, this study did not evaluate the impacts of COVID-19 co-infections with other viruses and medication interactions on adverse events due to data limitations. The lack of COVID-19 co-infection and drug interaction data represents another constraint of this analysis. Lastly, the calculation of the reporting odds ratio (ROR) is sensitive to individual values, and it may be unreliable when one of the theoretical frequencies in the 2 × 2 contingency table is small or the denominator is 0.

## 5 Conclusion

This pharmacovigilance study used the real FAERS database to assess adverse event risks associated with molnupiravir therapy in COVID-19 patients. However, further clinical studies are needed for confirmation.

Overall, besides common gastrointestinal disorders, skin and subcutaneous tissue disorders were also prevalent. The study found that compared to other RNA antiviral drugs such as remdesivir, molnupiravir demonstrated a lower risk of serious adverse events in individuals under 65 years old. Nonetheless, closer monitoring of drug safety is necessary for individuals ≥65 years old. The adverse events reported in FAERS data align with most clinical trial findings. Ongoing monitoring is vital as the use of molnupiravir increases, enabling a more comprehensive understanding of its safety profile. These findings provided invaluable real-world data for post-marketing surveillance of molnupiravir and impor€tant guidance for its future clinical use.

## Data Availability

Publicly available datasets were analyzed in this study. This data can be found here: https://fis.fda.gov/extensions/FPD-QDE-FAERS/FPD-QDE-FAERS.html.
